# Exploring the Antibacterial Potential of *Artemisia judaica* Compounds Targeting the Hydrolase/Antibiotic Protein in *Klebsiella pneumoniae*: In Vitro and In Silico Investigations

**DOI:** 10.3390/ph17060667

**Published:** 2024-05-22

**Authors:** Fahdah Ayed Alshammari

**Affiliations:** Department of Biology, College of Science, Northern Border University, Arar 76312, Saudi Arabia; fahdah.ayed@nbu.edu.sa

**Keywords:** antibacterial, *Artemisia judaica*, *Klebsiella pneumoniae*, GC/MS, protein, hydrolase/antibiotic, in vitro, in silico, in vivo

## Abstract

Carbapenem antibiotic resistance is an emerging medical concern. Bacteria that possess the *Klebsiella pneumoniae* carbapenemase (KPC) protein, an enzyme that catalyzes the degradation of carbapenem antibiotics, have exhibited remarkable resistance to traditional and even modern therapeutic approaches. This study aimed to identify potential natural drug candidates sourced from the leaves of *Artemisia judaica* (*A. judaica*). The phytoconstituents present in *A. judaica* dried leaves were extracted using ethanol 80%. A reasonable amount of the extract was used to identify these phytochemicals via gas chromatography/mass spectrometry (GC/MS). One hundred twenty-two bioactive compounds from *A. judaica* were identified and subjected to docking analysis against the target bacterial protein. Four compounds (PubChem CID: 6917974, 159099, 628694, and 482788) were selected based on favorable docking scores (−9, −7.8, −7.7, and −7.5 kcal/mol). This computational investigation highlights the potential of these four compounds as promising antibacterial candidates against the specific *K*PC protein. Additionally, in vitro antibacterial assays using *A. judaica* extracts were conducted. The minimum inhibitory concentration (MIC) against the bacterium *K. pneumonia* was 125 μg/mL. Well–disk diffusion tests exhibited inhibition zones ranging from 10.3 ± 0.5 mm to 17 ± 0.5 mm at different concentrations, and time–kill kinetics at 12 h indicated effective inhibition of bacterial growth by *A. judaica* leaf extracts. Our findings have revealed the pharmaceutical potential of *Artemisia judaica* as a natural source for drug candidates against carbapenem-resistant pathogens.

## 1. Introduction

The rise of carbapenem-resistant Enterobacteria represents a critical healthcare challenge, as it undermines the effectiveness of carbapenem antibiotics in the treatment of infections. This resistance poses significant difficulties in effectively managing patients and contributes to elevated rates of illness and mortality associated with bacterial infections [1]. One common resistance mechanism involves carbapenemase enzyme production [2]. The most commonly observed carbapenemases include OXA-48, KPC, NDM, and VIM types, although their prevalence varies significantly across countries and regions [3]. *Klebsiella pneumoniae* carbapenemase (KPC) is a class-A serine beta-lactamase discovered in the United States in 1996 [4]. In several areas, including India, the Mediterranean region, and some European countries, it has emerged as a common mechanism of carbapenem resistance among Enterobacteria [2,5]. Historically, KPC carbapenemases have demonstrated a broad spectrum of substrates, including penicillins, aztreonam, cephalosporins, and carbapenems. Notably, they exhibit resistance to commonly used beta-lactamase inhibitors such as sulbactam, clavulanic acid, and tazobactam [2,6,7,8].

The emergence of antibiotic-resistant strains has presented significant challenges in the management of bacterial infections, necessitating the development of novel therapeutic strategies. Discovering new antibiotics or alternative natural products, including plants, which have been used for centuries in traditional medicine, and improving the efficacy of existing ones through dosing regimens or synergy therapies are potential approaches [9]. Over the past three decades, extensive research has been conducted to investigate the antimicrobial properties of various plants, among which *Artemisia* has garnered significant attention [10]. Artemisia is a broad, diverse genus of plants that fall under the family Asteraceae, encompassing 200 to 400 species [11]. Artemisia species exhibit growth in temperate regions across both hemispheres, typically inhabiting arid or semiarid environments. Artemisia is a group of resilient herbaceous plants and shrubs renowned for their essential oils’ potent chemical components. These substances exhibit a range of biological actions, such as antioxidant, antibacterial, anticancer, digestive enzyme inhibitory, anti-inflammatory, anthelmintic, and numerous other properties [12].

*Artemisia judaica* L. is an aromatherapeutic plant abundant in Saint Katherine, Sinai, Egypt. Many bioactive compounds associated with plant secondary metabolites were found in the GC/MS analysis of Artemisia species. Some of these newly found phytochemical components show biological action [13]. *Artemisia* species are essential medical plants now attracting significant interest due to their bioactive compounds. The interest in them is primarily driven by their use in cosmetic products, manufacture of essential oils, chemical diversity, and biological functionality [13,14,15,16].

Due to its many advantageous qualities, such as its anti-inflammatory, antibacterial, and antidiabetic effects, it has long been used in traditional medicine. Furthermore, in different Arabic regions, including Saudi Arabia, it is frequently employed in folk medicine for addressing conditions such as fungal diseases, atherosclerosis, diabetes, cancer, arthritis, and inflammatory-related disorders, which are among the conditions that need attention [17,18,19]. Despite extensive research on the inhibitory effects of *A. judaica*, especially against specific organisms, there is a lack of information regarding its antibacterial properties against *K. pneumoniae* pathogens. Despite the use of *A. judaica*, its potential in Saudi Arabia remains largely unexplored, and comprehensive studies are scarce.

A global concern regarding antibiotic-resistant bacterial strains underscores the critical nature of this research. Although numerous investigations have been conducted into the antimicrobial properties of different plant species, the antibacterial potential of compounds found in *A. judaica* against *Klebsiella pneumoniae* has received relatively little attention. By filling this knowledge vacuum, the study intends to shed new light on the mechanisms by which these compounds operate. By integrating in vitro and in silico analyses, the research provides a holistic methodology for assessing the compounds’ antibacterial properties. The examination identified a cumulative sum of 110 constituents from the oils extracted from the entire *A. judaica* plant (including stems, foliage, and blossoms), and 81 constituents from the *A. herba-alba* plant. The oil of *A. judaica* contained β-eudesmol, hexadecanoic acid, spathulenol, eudesma-4 (15), 7-dien-1-β-ol, carvacrol, and thymol as its primary constituents. These constituents constitute 67.3% of the total composition of essential oils, equivalent to 96.7% of the oil itself [20].

Hence, the primary objective of this study was to investigate the antibacterial potential of compounds obtained from *A. judaica*, with a specific focus on their ability to target the hydrolase/antibiotic protein in *Klebsiella pneumoniae*. To accomplish this, a comprehensive approach was employed, combining various in vitro assays such as MIC, disk–well diffusion, and time–kill curve assays. Additionally, in silico investigations involved docking analysis and ADME assays. Through these experimental and computational methods, the effectiveness of *A. judaica* compounds against *Klebsiella pneumoniae* were assessed, aiming to gain valuable insights into their potential as antibacterial agents.

## 2. Results

### 2.1. Protein and Phytochemical Preparation

**Figure 1:**
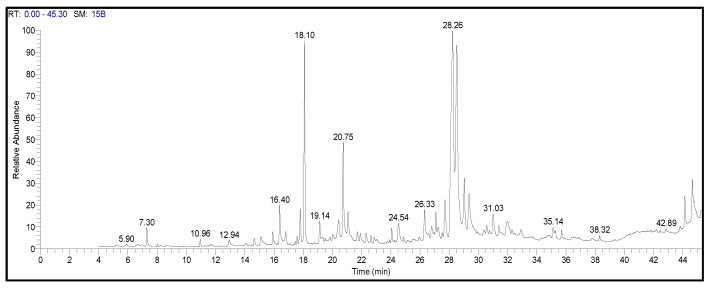
GC/MS chromatogram for the entire plant ethanolic extract of *Artemisia judaica.*

The study involved the extraction of chemical compounds from the plant *Artemisia judaica* using GC mass analysis, a widely used analytical technique (Figure 1). A total of 122 phytochemical compounds derived from the plant have been obtained and stored (Table S1). The compounds were synthesized and refined throughout the ligand production protocols and then transformed into the pdbqt file format for later assessment. The optimization and organization of the protein were conducted in preparation for the molecular docking approach using the AutoDock software PyRx-0.8 version. The compounds were then stored in the pdbqt file format in preparation for the docking. Table 1 presents a selection of four compounds based on their docking scores (kcal/mol) and corresponding details such as PubChem ID, chemical name, formula, and binding affinity.

### 2.2. Active Position Detection and Generation of Receptor Grids

**Figure 2:**
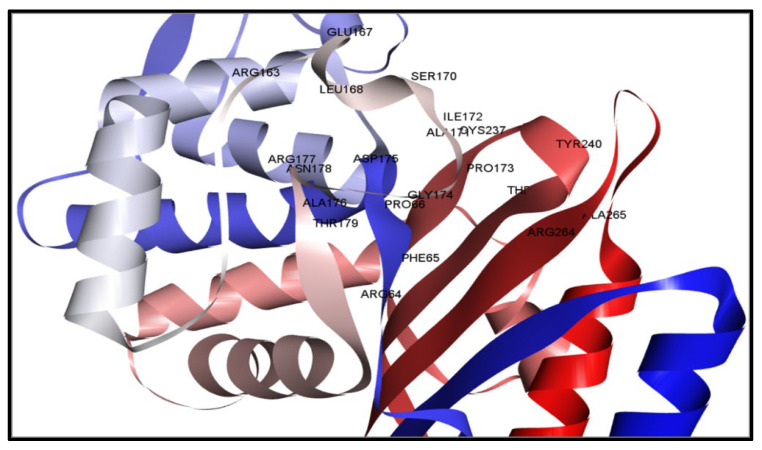
This image depicts the active site and the specific location where the protein forms a bond.

The configuration of many amino acid residues within a designated area gives rise to the active region of an enzyme, which facilitates transient contact with the substrate, generally referred to as the binding site. A chemical substrate can bind with the protein’s active site, leading to the catalysis of a process. Furthermore, it assists in stabilizing the intermediate stages of the process. In contrast, the concept of a binding site refers to a specific location on a protein or nucleic acid that has the potential to recognize a ligand and establish a robust binding association with the protein. The initial step in the research was to use the CASTpi service to find the protein’s active site (AS). After that, the active site’s mixed binding spot was found (Figure 2). By looking at the protein’s active site, it was easier to find the protein’s binding position residue (Figure 2). The study of the active site pocket identified the binding site locations at ARG64, PHE65, PRO66, ARG163, GLU167, LEU168, SER170, ALA171, ILE172, PRO173, GLY174, ASP175, ALA176, ARG177, ASN178, THR179, CYS237, TYR240, THR242, ARG264, and ALA265 residues. These positions are visually represented in Figure 2 using spherical shapes of varying colors, namely red, green, and orange. The server has discovered binding sites, which have been used to create a receptor grid in conducting a molecular docking simulation. The sizes of the grid box are X = 25Å, Y = 25Å, and Z = 43.54Å.

### 2.3. Evaluation of Protein and Ligand Interaction

**Figure 3:**
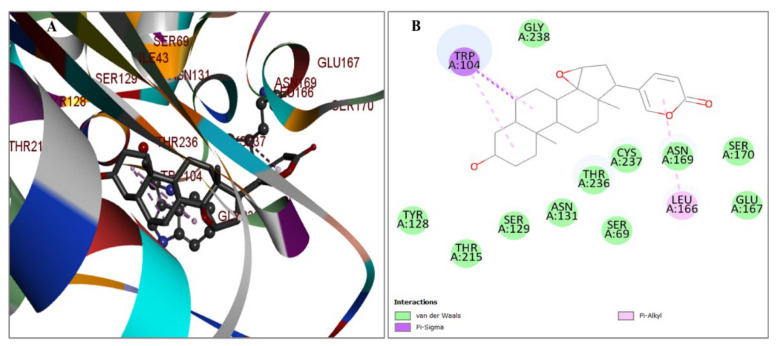
The interaction between the protein and the compound CID6917974. The two-dimensional bonding of the protein–ligand complex is depicted on the right (**B**), whereas the three-dimensional interaction is illustrated on the left (**A**). The chemical CID159099 was observed to establish numerous hydrogen and also carbon–hydrogen interactions with the preferred protein. The hydrogen bonding interactions occurring at the SER69, SER129, and ASN131 positions, together with the presence of single carbon–hydrogen bonds at the THR236 position, and TRP104 position hydrophobic bonds are depicted in Figure 4. The specific bond types are provided in Table 2.

Once the protein with the highest binding score was identified, a detailed analysis was undertaken to explore the intricate interactions between these substances. The research aimed to examine the utilization of the BIOVIA Discovery Studio Visualizer Tool 16.1.0, a software tool specifically designed for studying protein–ligand interactions, and the interaction between the four identified ligands and the target protein. The molecule CID6917974 has been shown to establish several hydrophobic interactions at the residue positions TRP104 and LEU166. The information in Figure 3 is documented in Table 2.

The compound CID628694 demonstrated a substantial affinity for the goal protein in relation to binding. The molecule has two hydrogen bonding interactions at SER69 and THR236 sites. On the other hand, three hydrophobic interactions occur at LEU166 and TRP104 positions. The data presented in Figure 5 are also documented in Table 2.

Compound CID482788 demonstrated an important level of affinity for the target protein in terms of binding. The molecule has hydrophobic interactions at position TRP104. The data presented in Figure 6 are also documented in Table 2.

### 2.4. ADME Calculation

The assessment of pharmacokinetics (PK) properties, which encompasses the evaluation of pharmaceuticals, including their kinetics (movement), is a crucial element in developing drug and procedure designs. However, the focus of analysis mainly lies in the ADME characteristics. The physiochemical parameters included in this study comprise lipophilicity and water solubility. The discussion consists of pharmacokinetics, drug similarity, and medicinal chemistry, which have been presented. There is one potential explanation for the selection of optimal medication candidates. Before administering medicines in preclinical analyses, assessing pharmacophore characteristics may ascertain whether the molecule exhibits characteristics related to the control of xenobiotics. The pharmacophore characteristics of the four chosen drug-like compounds should be examined, which require determination by lipids, which should be analyzed using the SwissADME program. A solvent that lacks a net dipole moment does not exhibit significant polarity. Pharmacophore characteristics have ultimately shown favorable outcomes with the chemical in question showing efficacy and potential for medication development in scientific research. The pharmacokinetic characteristics of the four selected drugs are illustrated in Table 3.

### 2.5. Antibacterial Activity

#### 2.5.1. MIC

The antibacterial effects of *A. judaica* extract against *K. pneumoniae* were examined in this research. The antibacterial activity of the extract was assessed by a combination of qualitative methods, such as well and disk diffusion assays, as well as a quantitative approach known as the minimum inhibitory concentration (MIC) test. The identification of bacterial proliferation was achieved by visually assessing the broth’s turbidity. The MIC of *A. judaica* extract against *K. pneumoniae* was 125 μg/mL (Figure 7). This value was observed at the fourth dilution.

#### 2.5.2. Well Diffusion Method

The inhibition zones measured by the well diffusion assay against the pathogenic bacterium *K. pneumoniae* were found to vary between 10.3 ± 0.5 mm and 17 ± 0.5 mm at 125, 250, and with sterile distilled water (SDW). In comparison, the effect of amoxicillin against *K. pneumoniae* was found to be 22.3 mm (Figure 8).

#### 2.5.3. Disk Diffusion Method

The findings from the disk diffusion test demonstrated that the extract derived from *A. judaica* exhibited inhibitory effects on the proliferation of harmful bacteria, as depicted in Figure 9. The inhibition zones against *K. pneumoniae* varied between 11 ± 0.5 mm and 14 ± 0.5 mm at doses of 125, 250, and 500 µg/mL, respectively. As a positive control, oxacillin 1 µg (OXC 1) exhibited an effect of 23.3 mm against *K. pneumoniae*.

#### 2.5.4. Time–Kill Curve

The time–kill curves of *Artemisia judaica* extract against *K. pneumoniae* were evaluated and associated with the control group using augmentin. The outcomes showed that the extract had a time-dependent antibacterial effect, gradually reducing the colony-forming units (CFUs) of *K. pneumoniae* over 24 h. Compared with the control group, the extract-treated group exhibited a decrease in CFUs at each time point (Figure 10). However, augmentin displayed a more rapid bactericidal effect, leading to the complete eradication of *K. pneumoniae* within 10 to 12 h.

## 3. Discussion

In the modern era, a multitude of research studies have extensively documented the remarkable efficacy of plant extracts in combating pathogenic bacteria that pose a threat to human health. These investigations have shed light on the potent antibacterial properties exhibited by diverse plant extracts [21,22,23]. In our investigation, we conducted both in silico and in vitro studies. A total of 122 trivial molecules were extracted from *A. judaica* using GC/MS analysis. It is important to note that of all the main chemicals identified in *A. judaica,* we showed only four docking scores and interactions using in silico methods. But in the crude extract, the docking score of some compounds was also high, such as of naphthalene (−7.2), nootkaton-11 (−7), stigmastero (−7), phthalic acid (−6.9), cis-sesquisabinene hydrate (−6.7), vulgarin (−6.7), (e)-isovalencenal (−6.5), methyl ester (−6.5), etc.

In contrast, while previous studies [17,24,25] have identified the phytoconstituents of *A. judaica*, it is important to note that these constituents can vary due to factors such as growing conditions, climatic conditions, cultivar, soil, plant age, and flowering state. Therefore, in this study, we analyzed the plant extract to examine its specific phytochemical composition. Through GC/MS analysis of the *A. judaica* extract obtained from its aerial parts, we detected 122 known compounds listed in S1 along with their chemical formulas. The phytochemical profiles of the extract revealed a high abundance of compounds in the aerial parts of *A. judaica*.

The utilization of computational or in silico methodologies is gaining significance in modern drug exploration attempts since they play a crucial role in promptly identifying promising drug candidates, often at a reduced expense compared to conventional techniques. Additionally, these methodologies reduce animal models in pharmacological investigations, facilitate the systematic development of innovative and secure drug candidates, repurpose pre-existing therapeutic agents, and aid medicinal chemists and pharmacologists in all drug exploration endeavors [26]. Therefore, identifying the possible protein of *K. pneumoniae* could serve as a viable target for rendering the protein associated with pathogenicity inactive. Given its distinctive characteristics, the crystal arrangement of the KPC-2 D179N variant (PDB: 8G2R) was selected as a prospective target for pharmaceutical intervention [27,28].

**Figure 4:**
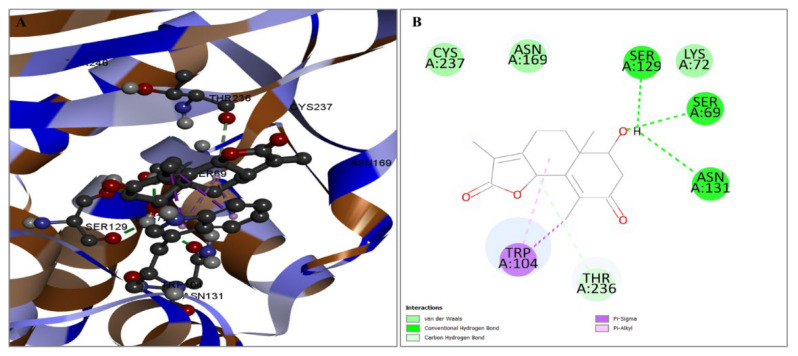
The relationship between the chemical CID159099 and the protein. The figure on the left depicts the 3D interaction (**A**), while the diagram on the right depicts the 2D contact between the protein and ligand compound (**B**).

**Figure 5:**
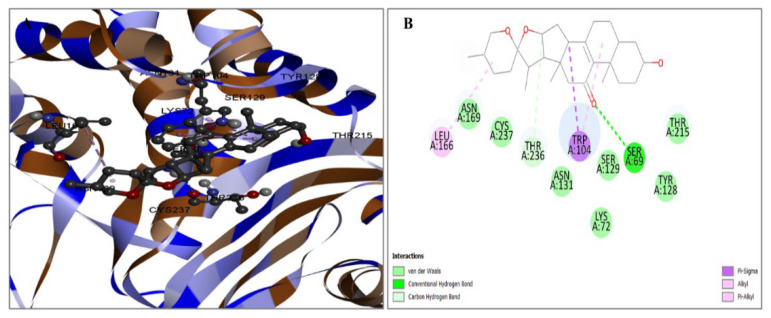
The interaction with protein and compound CID628694 is illustrated. The three-dimensional interaction is depicted on the left (**A**), while the protein–ligand complex’s two-dimensional interaction is illustrated on the right (**B**).

**Figure 6:**
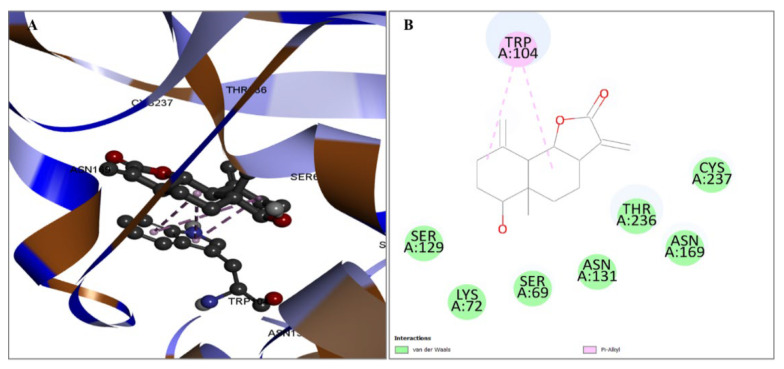
The right side represents the 2D interaction of the protein–ligand complex (**B**), whereas the left side is the 3D interaction of CID482788 (**A**).

**Figure 7:**
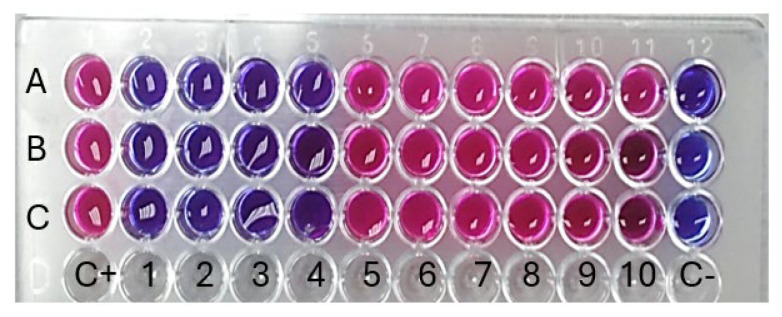
Resazurin dye was used to define A’s lowest inhibitory concentration (MIC)*. A. judaica* extracts against *K. pneumoniae*. Rows A–C in the dataset correspond to triplicate samples of the bacterial strain. Columns 1 through 10 depict the sequential dilution of *A. judaica* extract with medium, whereas column C+ serves as a positive control consisting solely of cultured strains. Column C-, on the other hand, solely includes media, representing a lack of plate contamination throughout the preparation process.

**Figure 8:**
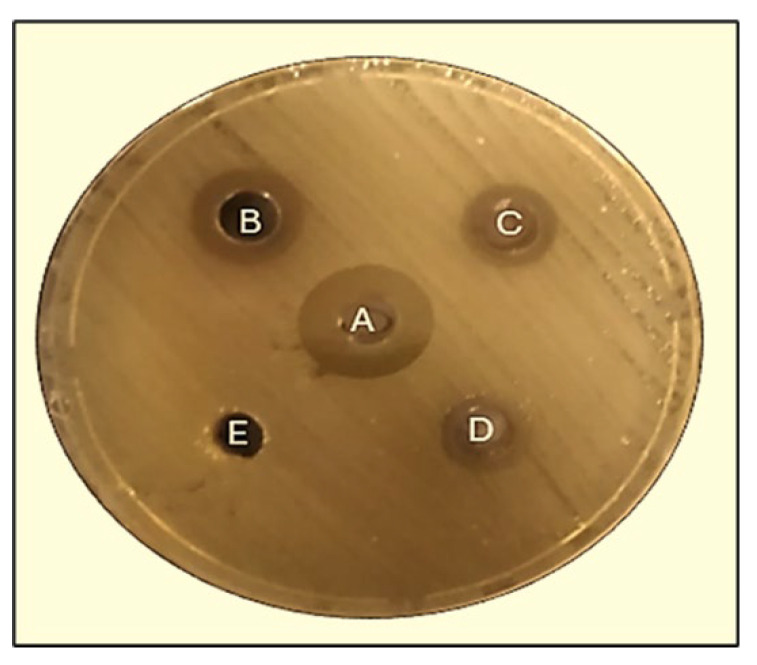
Zones of inhibition (mm) of *A. judaica* against *K. pneumoniae* as determined by agar well diffusion. A, Amoxicillin; B, 500 µg/mL; C, 250 µg/mL; D, 125 µg/mL; E, Sterile distilled water (SDW).

**Figure 9:**
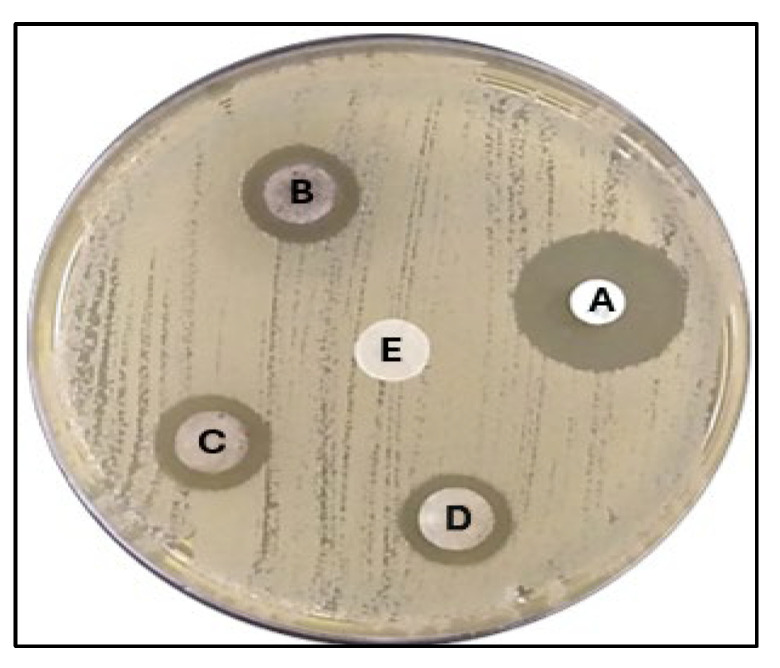
Zones of inhibition (mm) of *A. judaica* against *K. pneumoniae* as determined by agar disk diffusion. Oxacillin (A), 500 µg/mL (B), 250 µg/mL (C), 125 µg/mL (D), and sterile distilled water (SDW) (E).

**Figure 10:**
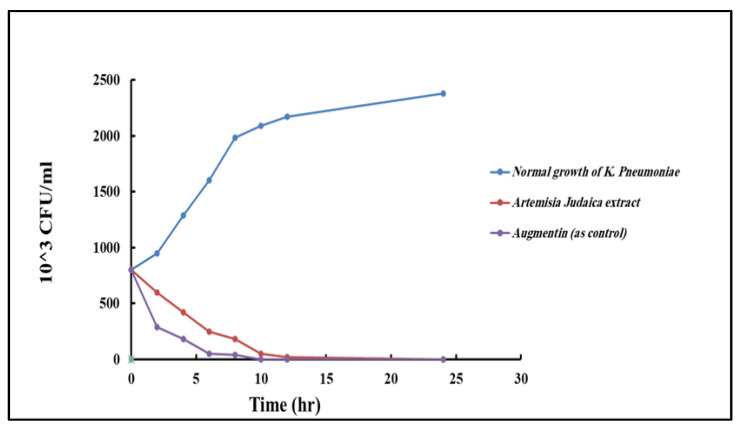
Time–kill curve for *K. pneumoniae* of *Artemisia judaica* extract and augmentin as control.

In this study, 122 compounds were subjected to docking using AutoDock Vina (Table S1). Four compounds were identified as promising candidates, and the most capable compounds were chosen for further simulation. Potential therapeutic candidates were found using various criteria (shown in Figure 2, Figure 3, Figure 4 and Figure 5). In addition, to ascertain the drug-like characteristics of the compounds, an analysis was conducted on their pharmacokinetic properties. The compounds also exhibited drug-like qualities, as shown in Table 3.

To evaluate the similarity of the identified phytoconstituents to established pharmaceuticals regarding their pharmacokinetics, toxicity, and physicochemical properties, a comparative study was conducted. The physicochemical parameters frequently examined in drug investigation encompass MW, LogS, HBA, HBD, nRTB, and LogP [29]. Wide-ranging studies have been conducted on these parameters to examine their impact on several pharmacokinetic factors, including absorption, bioavailability, permeability, and elimination, focusing on oral medications [30].

In vitro studies demonstrate the antibacterial efficacy of *Artemisia judaica* extract against *K. pneumoniae*, as evidenced by the observed inhibition zones and MIC values. Several studies have explored the antimicrobial properties of various *Artemisia* species, including *Artemisia judaica*, against different bacterial pathogens [18,31,32]. Some studies have reported similar findings, supporting the antibacterial efficacy of *Artemisia judaica* extract against pathogenic bacteria [33]. For example, a study on the antibacterial activity of *Artemisia* against a range of bacterial strains, including *K. pneumoniae*, was conducted and observed inhibition zones in agreement with the outcomes of the current study [34]. These findings suggest that *Artemisia judaica* extract possesses a broad-spectrum antibacterial effect. Variations could be due to changes in the extraction methods, plant sources, geographical locations, or even variations in the bacterial strains used in the studies. Furthermore, it is important to acknowledge that comparing findings across multiple studies might present difficulties owing to disparities in experimental parameters, including using distinct solvents, concentrations, and testing procedures. These factors can significantly impact the outcomes and make direct comparisons difficult. To further validate the findings of this study and establish the consistency of *Artemisia judaica* extract’s antibacterial efficacy against *K. pneumoniae*, additional studies involving larger sample sizes, standardized testing protocols, and rigorous statistical analysis are needed. Moreover, investigating the potential mechanisms of action and identifying the active compounds responsible for the observed antibacterial effects would provide valuable insights for future research and potential pharmaceutical applications.

## 4. Materials and Methods

### 4.1. Plant Material

The study comprised fresh leaves from *Artemisia judaica* in the research location of Jeddah, Saudi Arabia, in 2023. The Department of Biological Sciences, found within the Faculty of Science of King Abdulaziz University, has verified the genuineness of the plant. The *A. judaica* leaves were submerged in flowing tap water and washed with distilled water before processing. Subsequently, the plants were desiccated in a shaded place for 14 days at ambient temperature. After drying, the leaves were pulverized into a fine powder using an electric blender [35,36,37].

#### 4.1.1. Preparation of Crude Extracts

The fresh leaves of *A. judaica* were dried and finely ground using an electric blender. The ground leaves were then placed in a glass flask with ethanol (80%) and incubated for two days. During this time, the flask was placed on a shaker (Shaker SHO 1-D) and agitated at a speed of 150 rpm for 48 h at room temperature. Subsequently, the mixture was filtered to separate the liquid extract. The extracted substance was made more potent by letting it evaporate at 55 °C and low pressure using a spinning evaporator (Buchi Rotavapor R-114 and Waterbath B-480). After concentration, the dry extract obtained was measured using an ADAM.0001g electronic balance, and the result was evaluated. Finally, in our study, the extraction yield of the raw plant material was determined to be 0.85 g. To ensure proper preservation, the desiccated extract was stored in a visually opaque glass container and kept refrigerated at a temperature of 4 °C until it was utilized for further analysis [38,39].

#### 4.1.2. GS/MS Analysis

A Trace GC-TSQ mass spectrometer (Thermo Scientific, Austin, TX, USA) with a TG–5MS straight capillary column (30 m × 0.25 mm × 0.25 µm film thickness) was used to determine the chemicals in the samples. At first, the column oven was 50 °C. The temperature was raised by 5 °C every minute, then held for two minutes, until it hit 250 °C. Subsequently, the temperature was raised to 300 °C, maintaining a hold time of 2 min and a variable rate of 30 °C per minute. The temperature readings for the injector and MS transfer lines were consistently maintained at 270 °C and 260 °C, respectively. The carrier gas was helium, maintaining a consistent flow rate of 1 mL/min. The attenuated samples of 1 µL were autonomously delivered into the apparatus using the split mode of the Autosampler AS1300, in combination with gas chromatography (GC), with a solvent delay of 4 min. Full scan mode was utilized to acquire the EI mass spectra at 70 eV ionization voltages, encompassing a 50–650 *m*/*z* range. The temperature of the ion source was reduced to 200 °C. The ingredients were determined by a comparison study, where the mass spectra of the chemicals were compared to those acquired from the WILEY 09 and NIST 14 mass spectral databases [40].

### 4.2. Identifying and Preparing Proteins

The molecular targets associated with the *Klebsiella pneumoniae* gene were identified, and the Protein Data Bank (PDB) was used to obtain the X-ray crystallography structures of these proteins. The PDB structures contain water particles, cofactors, and metal ions [27]. However, these structures cannot provide detailed information on topologies, bond ordering, and formal atomic charges. The obtained PDB structures underwent the “prepare protein” procedure in Discovery Studio 4.0 software [41]. The isolation of the target proteins was achieved by removing water molecules, ligands, and other heteroatoms from the structure.

### 4.3. Detection of Active Sites

The receptor proteins’ binding sites were identified using the “receptor cavity technique” in Accelrys Discovery Studio 4.0 [42]. This study’s method helped identify the target receptor’s active areas. The identification process involved the analysis of the inhibitory qualities exhibited by the amino acid residues present inside the receptor’s binding region.

### 4.4. Model Enhancement and Confirmation

The protein’s 3-dimensional structure was uploaded to the internet platform “Galaxy Refine” on 20 January 2024. This was performed to enhance the organization of the protein. The quality of the utmost structure, energy score, and root mean square deviation (RMSD) value were determined utilizing the Galaxy Refine server [43]. The investigation required identifying the maximum and minimum root mean square deviation (RMSD) values. The ordinary interatomic distance and energy level were considered parameters while selecting the new structure. The PyMol v2.3.4 program displayed an image of the improved structure [44]. The fundamental value determines the standard deviation. The Z-score plot is available on the ProSA website at https://prosa.services.came.sbg.ac.at/prosa.php as of 25 January 2024.

### 4.5. Protein and Ligand Processing

Before docking, it is essential to fine-tune and verify the protein structures meticulously. The 3D framework of the protein was created using AutoDock Tools (ADT). The Gasteiger energy involves both the protein and the inclusion of non-polar hydrogen atoms. Moreover, it acts as a chelating agent for metallic ions and assists in removing cofactors from proteins. One hundred twenty-two chemicals from the medicinal plant *Artemisia judaica* were retrieved from the GC mass analysis. The compounds chosen for examination were sourced from a specified database and then underwent energy reduction utilizing the universal force field, which was considered specifically for the ligand molecules.

### 4.6. Finding Protein Active Positions and Generating Receptor Grids

The protein’s structural component has been uploaded to the CASTp 3.0 website, available at http://sts.bioe.uic.edu/. The proposal was completed on 27 January 2024. The web server recognized distinct regions within the protein and labeled the first one based on its surface area (SA). The identification and visualization of protein binding site residues were conducted using BIOVA Discovery Studio Visualizer Tool 16.1.0, facilitating the analysis of active pockets [45]. The receptor grids for molecular docking simulation were created using binding sites acquired from a web server, utilizing PyRx tools [46].

### 4.7. Simulation of Molecular Docking

Molecular docking was performed using PyRx-0.8 version software to conduct a simulated screening of the selected drugs. Virtual screening software has found several potential medications for different ailments [47]. This study integrates the docking capabilities of AutoDock along with AutoDock Vina into the Lamarckian genetic algorithm (LGA). Molecular docking interactions were conducted using the PyRx program AutoDock Vina [28]. BioVA Discovery Studio Visualizer Tools version 16.1.0.41 examines intricate key locations [48].

### 4.8. ADME Analysis

Evaluating a compound’s ADME characteristics is essential for developing potential medicinal qualities. Utilizing in silico methods to forecast the ADME properties of compounds in early drug development shows potential for decreasing the risk of failure in clinical trials. Various substances have been considered inappropriate for clinical trials and have not met market demand [49]. Therefore, ADME testing is a crucial element of the drug design process at its inception [50,51]. On 10 February 2024, the ADME web server was utilized to estimate the ADME features, including bioavailability, solubility profile, and gastrointestinal absorption of designated medications [52].

### 4.9. Bacterial Strain

The bacterial strain *K. pneumoniae* ATCC (13883), maintained in the department, was utilized as the bioreporter in this study. The isolate that had been kept was harvested from the glycerol stock and subsequently cultured in nutrient broth (NB) medium for further use. The initial culture was after that subjected to propagation under agitation conditions of 150 g at a temperature of 30 °C for a total duration of 24 h.

### 4.10. Antimicrobial Activity

#### 4.10.1. Estimation of Minimum Inhibition Concentration (MIC)

The minimal inhibitory concentration (MIC) of the A. judaica extract was determined using 96-well microtiter plates and the micro-broth dilution method. The extract was systematically diluted over a concentration range of 1000 µg/mL to 0.0031 µg/mL and exposed to incubation with the microorganisms at 37 °C for 24 h. Following the incubation period, the Resazurin dye was introduced to evaluate bacterial proliferation. To ensure accuracy and reliability, control columns were included in the experiment to validate the obtained results [53].

#### 4.10.2. Disk Diffusion Method

The bacterial strains were inoculated into Mueller–Hinton agar (MHA) media at 10^6^ colony-forming units per milliliter (cfu/mL). The *A. judaica* extract was then impregnated with three different concentrations (125, 250, and 500 µg/mL), and a 7 mm paper strainer disk was placed on the agar after being impregnated. Subsequently, the plant extract was allowed to undergo diffusion into the medium for 30 min at ambient temperature. Oxacillin (OXA) was used as a positive control and sterile distilled water (SDW) was the negative control. A temperature of 37 °C was maintained for the incubation period of 24 h on the plates. The zone of inhibition was calculated using the mean and standard deviation (SD) of triplicate experiments.

#### 4.10.3. Well Diffusion Method

The microbe was inoculated onto Mueller–Hinton agar (MHA) media at 106 colony-forming units per milliliter. A sterilized cork borer was used to create five holes in each culture plate. A positive control was prepared by adding 100 μL of amoxicillin, and a negative control was established using sterile distilled water (SDW). In the remaining three holes, 100 μL of Artemisia judaica extract was added at varying concentrations of 125, 250, and 500 µg/mL. For 24 h, the plates were placed in an incubator set at 37 °C. The zone of inhibition was quantified using the mean ± standard deviation (SD) of experiments conducted in triplicate.

#### 4.10.4. Time–Kill Curve

The time–kill kinetics were assessed using Mueller–Hinton broth to determine the time–kill kinetics. Augmentin was employed as a control in this study. First, 500 µg/mL *A. judaica* extract and 10 µg/mL augmentin solutions were introduced into the wells to determine time–kill kinetics. Microorganisms were exposed to aerobic incubation at a temperature of 37 degrees Celsius. The experimental protocol involved subjecting the samples to incubation at different intervals (0, 2, 4, 6, 8, 10, 12, and 24 h) in the wells containing the plant extract and the antibiotic solution. A sterile loop collected samples from the cultures, which were then evenly spread on blood agar plates. Subsequently, the plates were subjected to incubation over 24 h at a temperature of approximately 37 °C. The quantification of bacterial clusters in the cultures was performed using a plate counter after the incubation period [54].

## 5. Conclusions

The present research conducted both in silico and in vitro studies to investigate the antibacterial prospects of an ethanolic crude extract obtained from *Artemisia judaica* collected in the Jeddah region of Saudi Arabia against *K. pneumoniae*. Unfortunately, effective therapeutics targeting *K. pneumoniae* infections are currently unavailable, leading to a high incidence of *K. pneumoniae* infections. Consequently, this research aimed to explore potential natural and potent antibacterial agents against *K. pneumoniae.* To achieve this, computer-aided drug design (CADD) methods were employed, involving molecular docking and simulation of ADMET methods. This study investigated the antibacterial properties of *Artemisia judaica* extract against *K. pneumoniae* using qualitative and quantitative methods. Through these techniques, four potential antibacterial candidates, namely resibufogenin (CID6917974), naphtho(1,2-b)furan-2,8(3H,4H)-dione, 3a,5,5a,6,7,9b-hexahydro-6-hydroxy-5a,9-dimethyl-3-methylene-,(3aS,5aR,6R,9bS) (CID159099), 16-hydroxy-5′,7,9,13-tetramethylspiro[5-oxapentacyclo[10.8.0.02,9.04,8.013,18]icos-1(12)-ene-6,2′-oxane]-11-one (CID628694), and reynosin (CID482788), were identified against *K. pneumoniae*.

The extract showed a minimum inhibitory concentration (MIC) of 125 μg/mL, inhibiting bacterial growth. In well and disk diffusion assays, the extract exhibited inhibition zones ranging from 10.3 ±0.5 mm to 17 ± 0.5 mm at different concentrations. Time–kill curve analysis demonstrated a gradual reduction in *K. pneumoniae* colony-forming units (CFUs) over 24 h, indicating a time-dependent antibacterial effect. These findings suggest the potential of *Artemisia judaica* extract as an alternative treatment against *K. pneumoniae*, warranting further investigation. However, further investigation, particularly in vivo studies, is recommended to evaluate the efficiency of these compounds against *K. pneumoniae* strains causing diseases.

## Figures and Tables

**Table 1:** A list of four compounds was chosen based on their docking of molecules score (kcal/mol) and PubChem ID, chemical name, formula, and binding affinity.

PubChem CID	Chemical Name	Formula	Binding Affinity (Kcal/mol)
**6917974**	Resibufogenin	C_24_H_32_O_4_	−9.0
**159099**	Naphtho(1,2-b)furan-2,8(3H,4H)-dione, 3a,5,5a,6,7,9b-hexahydro-6-hydroxy-5a,9-dimethyl-3-methylene-,(3aS,5aR,6R,9bS)	C_15_H_18_O_4_	−7.8
**628694**	16-hydroxy-5′,7,9,13-tetramethylspiro[5-oxapentacyclo[10.8.0.02,9.04,8.013,18]icos-1(12)-ene-6,2′-oxane]-11-one	C_27_H_40_O_4_	−7.7
**482788**	Reynosin	C_15_H_20_O_3_	−7.5

**Table 2:** The chart of the connection and relationships of four designated phytochemicals with protein.

PubChem CID	Residue	Distance	Category	Type
**CID6917974**	TRP104	3.69752	Hydrophobic	Pi-Sigma
	TRP104	4.65974	Hydrophobic	Pi-Alkyl
	TRP104	4.07224	Hydrophobic	Pi-Alkyl
	TRP104	5.46292	Hydrophobic	Pi-Alkyl
	LEU166	5.42357	Hydrophobic	Pi-Alkyl
**CID159099**	SER69	2.35134	Hydrogen Bond	Conv-H-Bond
	SER129	2.06128	Hydrogen Bond	Conv-H-Bond
	ASN131	2.92787	Hydrogen Bond	Conv-H-Bond
	THR236	3.51664	Hydrogen Bond	Carbon Hydrogen Bond
	TRP104	3.50246	Hydrophobic	Pi-Sigma
	TRP104	3.85502	Hydrophobic	Pi-Sigma
	TRP104	5.23469	Hydrophobic	Pi-Alkyl
	TRP104	4.48891	Hydrophobic	Pi-Alkyl
**CID628694**	SER69	3.01252	Hydrogen Bond	Conv-H-Bond
	THR236	3.68071	Hydrogen Bond	Carbon Hydrogen Bond
	TRP104	3.41301	Hydrophobic	Pi-Sigma
	LEU166	5.36347	Hydrophobic	Alkyl
	TRP104	4.06557	Hydrophobic	Pi-Alkyl
	TRP104	5.18272	Hydrophobic	Pi-Alkyl
**CID482788**	TRP104	5.13792	Hydrophobic	Pi-Alkyl
	TRP104	4.22344	Hydrophobic	Pi-Alkyl
	TRP104	4.44737	Hydrophobic	Pi-Alkyl
	TRP104	5.06342	Hydrophobic	Pi-Alkyl

**Table 3:** The structures of ADME of the four drugs are included in the list of pharmacokinetics. The lists also show many physicochemical characteristics of those compounds and also include distinct physicochemical parameters of the substances.

Properties		CID6917974	CID159099	CID628694	CID482788
**Phytochemical properties**	MW (g/M)	384.51	262.30	428.60	248.32
	Heavy atoms	28	19	31	18
	Aromatic heavy atoms	6	0	0	0
	Rotatable bond	1	0	0	0
	Hydrogen bond acceptors	4	4	4	3
	Hydrogen-bond donor	1	1	1	1
**Lipophilicity**	Log Po/w	3.88	1.64	4.30	2.34
**Water solubility**	Log S (ESOL)	−4.67 (moderately soluble)	−1.98 (soluble)	−5.14 (moderately soluble)	−2.66 (soluble)
**Pharmacokinetics**	GI absorption	High	High	High	High
**Drug-likeness**	Lipinski	Yes (0 violations)	Yes (0 violations)	Yes (0 violations)	Yes (0 violations)
**Medicinal chemistry synthesis accessibility**		5.98	4.41	6.70	4.06

## Data Availability

Data are contained within the article.

## Supplementary Materials

The following supporting information can be downloaded at: https://www.mdpi.com/article/10.3390/ph17060667/s1, Table S1: GC mass result of *A. judaica*.
