# Supplementary material for: Exploring the Antibacterial Potential of Artemisia judaica Compounds Targeting the Hydrolase/Antibiotic Protein in Klebsiella pneumoniae: In Vitro and In Silico Investigations
Source: Pharmaceuticals (Basel). 2024 May 22;17(6):667. doi: 10.3390/ph17060667 (PMC11207000; doi:10.3390/ph17060667)
Supplement: Supplementary file 1 [file pharmaceuticals-17-00667-s001.zip › pharmaceuticals-2963291-supplementary.pdf]

Stable 1: GC mass result *of A. judaica*

Sl	RT	Compound name	Formula	Structure	MW	Peak Area%
1	7.29	Camphor	C10H16O		152	1.17
2		1,7,7-TRIMETHYL-BICYCLO[2.2.1]HEPTAN-2-ONE				
3		+)-2-Bornanone				
4		Bicyclo[2.2.1]heptan-2-one,1,7,7-trimethyl-, (1S)-				
5	10.96	BICYCLO[2.2.1]HEPTAN-2-OL,1,7,7-TRIMETHYL-, ACETATE,(1S-ENDO)-	C12H20O2		196	0.46
6		Bornyl acetate				
7		1,7,7-trimethyl-bicyclo[2.2.1]hept-2-yl ester				
8	14.65	trans-p-mentha-1(7),8-dien-2-ol	C10H16O		152	0.5
9		7-epi-trans-sesquisabinene hydrate				
10		Bicyclo[3.1.1]hept-2-en-4-ol,2,6,6-trimethyl-, acetate				
11		4,6,6-TRIMETHYLBICYCLO[3.1.1]HEPT-3-EN-2-YL ACETATE				
12		7-epi-cis-sesquisabinene hydrate				
13	15.1	2-Propenoic acid, 3-phenyl-, ethyl ester, (E)-	C11H12O2		176	0.65
14		2-PROPENOIC ACID, 3-PHENYL-,ETHYL ESTER				
15		2-Propenoic acid, 3-phenyl-, ethyl ester				
16		Ethyl (Z)-cinnamate				
17	15.93	DAVANA ETHER 1	C15H22O2		234	0.81
18		Davana ether				
19		FURAN,5-[1-(5,5-DIMETHYL-2(5H)-FURA NYLIDENE)ETHYL]-2-ETHENYL TETRAHYDRO-2-M ETHYL-				
20		DAVANA ETHER 2				
21	17.6	5-Hepten-3-one,2-(5-ethenyltetrahydro-5-methyl-2-furanyl)-6-methyl-, [2S-[2à(R*),5à]]	C15H24O2		236	0.46
22	17.81	1,6,10-Dodecatrien-3-ol,3,7,11-trimethyl-, (E)-	C15H26O		222	2.22
23		Nerolidol				
24	19.14	Cyclopentaneacetic acid,3-oxo-2-(2-pentenyl)-, methyl ester,[1à,2à(Z)]	C13H20O3		224	1.45
25		METHYL (3-OXO-2-[(2Z)-2-PENTENYL]CYC LOPENTYL)ACETATE #				
26		(3-OXO-2-PENT-2-ENYL-CYCLO PENTYL)-ACETIC ACID METHYL ESTER				
27		Methyl jasmonate				

28		CYCLOPENTANEACETIC ACID, 3-OXO-2-(2-PENTENYL)-,METHYL ESTER, (Z)-TRANS-				
29	20.45	E-8-Methyl-9-tetradecen-1-ol acetate	C17H32O2		268	1.82
30		NEROLIDOL-EPOXYACETATE	C17H28O4		296	
31		3-OXO-20-METHYL-11-à-HYDRO XYCONANINE-1,4-DIENE	C22H31NO2		341	
32	21.08	Chamazulene	C14H16		184	1.77
33		AZULENE,7-ETHYL-1,4-DIMETHYL-				
34		Naphthalene, 1,2,3,4-tetramethyl-				
35		1,2,3,4-TETRAMETHYLNAPHTHA LENE				
36	21.72	9-(3,3-Dimethyloxiran-2-yl)-2,7-dim ethylnona-2,6-dien-1-ol	C15H26O2		238	0.65
37		9-(3,3-DIMETHYL-2-OXIRANYL)-2,7-DIMETHYL-2,6-NONADIEN-1-OL				
38	21.93	2-((2R,4aR,8aS)-4a-Methyl-8-methyl enedecahydronaphthalen-2-yl)prop-2 -en-1-ol	C15H24O		220	0.58
39		(E)-2-((8R,8aS)-8,8a-Dimethyl-3,4,6,7,8,8a-hexahydronaphthalen-2(1H)-y lidene)propan-1-ol				
40		Cedren-13-ol, 8-				
41		Aristol-1(10)-en-9-ol				
42		Ledene oxide-(II)				
43	22.32	cis-5,8,11,14,17-Eicosapentaenoic acid	C20H30O2		302	0.74
44		9-t-Butyltricyclo[4.2.1.1(2,5)]decane-9,10-diol			224	
45		9-TERT-BUTYL-TRICYCLO[4.2.1.1 2,5]DECANE-9,10-DIOL			224	
46		ETHYL(9Z,12Z)-9,12-OCTADECADIENO ATE #			308	
47		Z,Z,Z-4,6,9-Nonadecatriene	C19H34		262	
48	24.08	Ethanol, 2-(9-octadecenyloxy)-, (Z)-	C20H40O2		312	0.93
49		ETHANOL,2-(9-OCTADECENYLOXY)-, (Z)-	C20H40O2		312	
50		3,7,11,15-Tetramethyl-2-hexadecen-1-ol	C20H40O		296	
51		7-Heptadecyne, 1-chloro-	C17H31Cl		270	
52		7-HEPTADECYNE, 1-CHLORO-	C17H31Cl		270	
53	24.54	Lidocaine	C14H22N2O		234	1.64
54		Ethanamine,N,N-diethyl-2,2-dimethyl-2-[(3,5-dimethoxy)phenyl]-	C16H27NO2		265	
55		2-(3,5-DIMETHOXYPHENYL)-N,N-DIETHYL-2-METHYL-1-PROP ANAMINE	C16H27NO2		265	
56		(3S,3AR,5S,8AR,9AR)-3-({[2-(DIETHYLAMINO)ETHYL]AMINO}METHYL)-5,8A-DIMETHYL-3A,5,6,7,8,8A,9,9A-OCTAHYDRONAPHTHO[2,3-B]FURAN-2(3H)-ONE	C21H36N2O2		348	

57		4-ethyl Pentedrone	C21H36N2O2		219	
58	26.33	HEXADECANOIC ACID	C16H32O2		256	2.15
59		n-Hexadecanoic acid				
60	26.8	Hanphyllin	C15H20O3		248	0.98
61		TRICYCLO[8.6.0.0(2,9)]HEXADECA-3,16,HEAD,HEAD-DIONE,CIS-2,9-TRANSOD-9,10-CIS-1,10	C16H24O2			
62		TRICYCLO[8.6.0.0E2,9]HEXADECA-3,16,KOPF,KOPF-DIONE,CIS-2,9-TRANSOD-9,10-CIS-1,10	C16H24O2			
63		TRICYCLO[8.6.0.0E2,9]HEXADECA-8,16,KOPF,SCHWANZ-DION,TRANS-2,9-CISOID-9,10-CIS-1,10	C16H24O2			
64		2,5-CYCLOHEXADIENE-1,4-DIONE,2,5-BIS(1,1-DIMETHYLPROPYL)-	C16H24O2			
65	27.27	Reynosin	C15H20O3		248	0.67
66		Azuleno[4,5-b]furan-2(3H)-one,decahydro-7,9-dihydroxy-6,9a-dimethyl-3-methylene-,[3aS-(3aà,6á,6aà,7à,9à,9aá,9bà)]-	C15H22O4		266	
67		1,4-Hexadien-3-one,5-methyl-1-[2,6,6-trimethyl-2,4-cyclohexadien-1-yl]	C16H22O		230	
68		2-(4a,8-Dimethyl-7-oxo-1,2,3,4,4a,7-hexahydronaphthalen-2-yl)-propionic acid	C15H20O3		248	
69		2-(4A,8-DIMETHYL-7-OXO-1,2,3,4,4A,7-HEXAHYDRO-2-NAPHTHALENYL)PROPANOIC ACID	C15H20O3		248	
70	27.72	LACTAROPALLIDIN	C15H24O3		252	3.02
71		AZULENO[4,5-B]FURAN-2(3H)-ONE,DECAHYDRO-8,9-DIHYDROXY-6,9A-DIMETHYL-3-METHYLENE-,[3AS-(3Aà,6á,6Aà,8à,9à,9Aá,9Bà)]-	C15H22O4		266	
72		7,10,13-Eicosatrienoic acid, methylester	C21H36O2		320	
73		Spiro[4.5]decan-7-one,1,8-dimethyl-8,9-epoxy-4-isopropyl-	C15H24O2		236	
74		SPIRO[4,5]DECAN7-ONE,1,8-DIMETHYL-8,9-EPOXY-4-ISOPROPYL-	C15H24O2		236	
75	28.27	NAPHTHO[1,2-B]FURAN-2,6(3H,4H)-DIONE,3A,5,5A,9,9A,9B-HEXAHYDRO-9-HYDROXY-3,5A,9-TRIMETHYL-,[3S-(3à,3Aà,5Aá,9à,9Aà,9Bá)]-	C15H20O4		264	20.84
76		Naphtho[1,2-b]furan-2,6(3H,4H)-dione,3a,5,5a,9,9a,9b-hexahydro-9-hydroxy-3,5a,9-trimethyl-				
77		NAPHTHO[1,2-B]FURAN-2,6(3H,4H)-DIONE3A,5,5A,9,9A,9B-HEXAHYDRO-9-HYDROXY-3,5A,9-TRIMETHYL-,[3S-(3à,3Aà,5Aá,9à,9Aà,9Bá)]-				

78	28.55	16-Methyloxacyclohexadeca-3,5-dien-2-one	C16H26O2		250	12.79
79		16-METHYL-OXA-CYCLOHEXADECA-3,5-DIEN-2-ONE	C16H26O2		250	
80		Thunbergol	C20H34O		290	
81		2(3H)-Benzofuranone,6-ethenylhexahydro-3,6-dimethyl-7-(1-methylethenyl)-,[3S-(3à,3aà,6à,7á,7aá)]-	C15H22O2		234	
82		Aromadendrene oxide-(2)	C15H24O		220	
83	29.06	1-Heptatriacotanol	C37H76O		536	3.45
84		01297107001 TETRANEURIN - A -DIOL	C15H20O5		280	
85		2-HEXADECEN-1-OL,3,7,11,15-TETRAMETHYL-,[R-[R*,R*-(E)]]-	C20H40O		296	
86		Phytol	C20H40O		296	
87		PHYTOL ISOMER	C20H40O		296	
88	2.38	Reynosin	C15H20O3		248	2.66
89		3H-Naphtho[2,3-b]furan-2-one,4-hydroxy-4a,5-dimethyl-3-methylene-3a,4,4a,5,6,7,9,9a-octahydro-	C15H20O3		248	
90		3H-NAPHTHO[2,3-B]FURAN-2-ONE,4-HYDROXY-4A,5-DIMETHYL-3-METHYLENE-3A,4,4A,5,6,7,9,9A-OCTAHYDRO-Androstan-17-one,3-ethyl-3-hydroxy-, (5à)-	C15H20O2		318	
91	31.03	Cycloisolongifolene,8,9-dehydro-9-vinyl-	C17H24		228	1.67
92		CYCLOISOLONGIFOLEN,8,9-DEHYDRO-9-VINYL-	C17H24		228	
93		8,9-DEHYDRO-9-VINYL-CYCLOISOLONGIFOLENE	C17H24		228	
94		ETHANONE,2-(2,2,4,6,6-PENTAMETHYLCYCLOHEXYLIDENE)-1-PHENYL-	C19H26O		270	
95		1-PHENANTHRENECARBOXYLIC ACID,1,2,3,4,4A,9,10,10A-OCTAHYDRO-6-HYDROXY-1,4A-DIMETHYL-,METHYL ESTER,[1S-(1à,4Aà,10Aá)]-	C18H24O3		288	
96	31.42	1-Heptatriacotanol	C37H76O		536	0.72
97		2-ACETYL-3-(2-CINNAMIDO)ETHYL-7-METHOXYINDOLE	C22H22N2O3		362	
98		Cholestan-3-ol, 2-methylene-,(3á,5à)-	C28H48O		400	
99		Dasycarpidan-1-methanol, acetate(ester)	C28H48O		326	
100	32.02	PREGNAN-20-ONE,5,6-EPOXY-3,17-DIHYDROXY-16-METHYL-, (3á,5à,6à,16à)-	C22H34O4		362	2.38

101		SPIRO[FURAN-2(5H),2'(1'H)-NAPHTHO[2,1-B]FURAN]-5-ONE,3'A,4',5',5'A,6',7',8',9',9'A,9'B-DECAHYDRO-3,3'A,6',6',9'A-PENTAMETHYL-,[2'R-(2'à,3'Aá,5'Aà,9'Aá,9'Bà)]-	C20H30O3		318	
102		Resibufogenin	C20H30O3		384	
103		Pregnan-20-one,3-(acetyloxy)-5,6-epoxy-17-hydroxy-16-methyl-,(3á,5à,6à,16à)-	C24H36O5		404	
104	35.14	PROPANOIC ACID, 2-METHYL-,(DECAHYDRO-6A-HYDROXY-9A-METHYL-3-METHYLENE-2,9-DIOXOAZULENO[4,5-B]FURAN-6-YL)METHYL ESTER,[3AS-(3Aà,6á,6Aà,9Aá,9Bà)]-	C19H26O6		350	1.08
105		N'-(2-METHYL-4-CHLOROPHENYL)-N-CYCLOHEXYFORMAMIDINE	C11H16N2		176	
106		PROPANOIC ACID, 2-METHYL-,(DECAHYDRO-6A-HYDROXY-9A-METHYL-3-METHYLENE-2,9-DIOXOAZULENO[4,5-B]FURAN-6-YL)METHYL ESTER,[3AS-(3Aà,6á,6Aà,9Aá,9Bà)]-	C19H26O6		350	
107		Nootkaton-11,12-epoxide	C15H22O2		234	
108	35.3	2-ACETYL-3-(2-CINNAMIDO)ETHYL-7-METHOXYINDOLE	C22H22N2O3		362	0.5
109		HAHNFETT				
110		Oleic acid, eicosyl ester	C38H74O2		562	
111	35.72	3',8,8'-Trimethoxy-3-piperidyl-2,2'-binaphthalene-1,1',4,4'-tetrone	C28H25NO7		487	0.57
112		2,6-DIMETHYL-N-(2-METHYL-à-PHENYLBENZYL)ANILINE	C22H23N		301	
113		9-(2',2'-Dimethylpropanoilhydrazono)-3,6-dichloro-2,7-bis-[2-(diethylamino)-ethoxy]fluorene	C30H42Cl2N4O3		576	
114		1,2-BENZENEDICARBOXYLIC ACID	C24H38O4		390	
115		PROMECARB 2,4-DINITROPHENYLETHER	C16H16N2O5		316	
116	44.17	Stigmasterol	C29H48O		412	2.22
117		STIGMASTA-5,22-DIEN-3-OL				
118		STIGMASTA-5,22-DIEN-3-OL,(3á,22E)-				

119	44.29	(22S)-6à,11á,21-Trihydroxy-16à,17à-propylmethylenedioxypregna-1,4-diene-3,20-dione	C25H34O7	446		0.74
120		6B-GLYCOLOYL-5,12-DIHYDROXY-4A,6A-DIMETHYL-8-PROPYL-4A,4B,5,6,6A,6B,9A,10,10A,10B,11,12-DODECAHYDRO-2H-NAPHTHO[2',1':4,5]INDENO[1,2-D][1,3]DIOXOL-2-ONE #	C25H34O7	446		
121		(22R)-6á,11á,21-Trihydroxy-16à,17à-propylmethylenedioxypregna-1,4-diene-3,20-dione	C25H34O7	446		
122		Spirost-8-en-11-one, 3-hydroxy-,(3á,5à,14á,20á,22á,25R)-	C27H40O4	428		
